# Coexistence of Autoimmune Pulmonary Alveolar Proteinosis With Small Cell Lung Cancer in a Man Presenting With Acute Onset Respiratory Failure

**DOI:** 10.7759/cureus.48330

**Published:** 2023-11-05

**Authors:** Ourania Papaioannou, Electra Koulousousa, Theodoros Karampitsakos, Argyrios Tzouvelekis

**Affiliations:** 1 Department of Respiratory Medicine, University Hospital of Patras, Patras, GRC

**Keywords:** sagramostim, inhaled gm-csf, whole lung lavage, sclc, apap

## Abstract

A 60-year-old man was referred to our Respiratory Department with progressive dyspnea on exertion and productive cough over the past two months. High-resolution computed tomography showed diffuse ground glass opacities with superimposed interlobular and intralobular septal thickening mainly in the middle and lower lobes, compatible with crazy paving pattern. Serology tests revealed positive antibody transcriptional intermediary factor-γ1 (TIF-1γ) in myositis panel and bronchoalveolar lavage revealed milky appearance and positive periodic acid-Schiff (PAS) stain. Pulmonary function tests showed moderate reduction in diffusing capacity for carbon monoxide. The working diagnosis of autoimmune pulmonary alveolar proteinosis was established by high detectable levels of anti-granulocyte-macrophage colony-stimulating factor (GM-CSF) antibodies. Despite clinical and radiological improvement following treatment with whole lung lavage and inhaled sargramostim, patient’s follow-up chest computed tomography revealed an enlargement of lower left paratracheal lymph node 4L. Endobronchial ultrasound bronchoscopy (EBUS) biopsy was compatible with small cell lung cancer (SCLC). Chemotherapeutic agents were promptly administrated, with no adverse events up until now.

## Introduction

Pulmonary alveolar proteinosis (PAP) is a rare disease characterized by alveolar accumulation of surfactant and progressive hypoxemia [1,2]. PAP is clinically divided into three different categories based on pathogenetic mechanisms; primary PAP that is caused by the disruption of granulocyte-macrophage colony-stimulating factor (GM-CSF) signalling and can be either autoimmune (characterized by elevated levels of GM-CSF autoantibodies) or hereditary (due to mutations in CSF2RA or CSF2RB that encode GM-CSF receptor subunits), secondary PAP resulting from various underlying conditions such as lysinuric protein intolerance, acute silicosis, inhalation of dusts (both organic and inorganic), immunodeficiency, malignancy and hematopoietic disorders and congenital PAP that is provoked by mutations in genes involved in surfactant production [3]. Autoimmune PAP (aPAP) is the most frequent form of PAP, occurring in 90% of cases with a prevalence of 7-10 per million [4]. Clinical and radiological presentation includes progressive dyspnea, cough, white sputum and infiltrates compatible with crazy paving pattern. Fever and hemoptysis may be indicative of intercurrent infection. In some cases, clinical course includes pulmonary fibrosis and respiratory failure. Recent research has established GM-CSF as a regulatory molecule critical to surfactant homeostasis, alveolar stability, lung function and host defense. Consequently, this data has raised aPAP from obscurity to a paradigm of molecular pathogenesis-based diagnostic and therapeutic development. Although whole-lung lavage remains the standard of care in therapy, inhaled GM-CSF is a promising safe, well tolerated and efficacious therapeutic modality [5,6]. With regards to lung malignancy as comorbidity, based on literature data, there are few case reports of aPAP associated with non-small cell lung cancer and only a case associated with small cell lung cancer (SCLC) diagnosed in postmortem anatomic pathological examination [7].

We herein report a rare case of autoimmune pulmonary alveolar proteinosis in the setting of small cell lung cancer in a middle-aged man presented with acute onset respiratory failure. Clinical vigilance and thorough diagnostic evaluation led to early diagnosis of the disease and timely therapeutic interventions. The following report presents in detail the patient’s management and clinical course, as well as highlights the cardinal role of close follow-up in management of rare clinical entities.

## Case presentation

**Figure 1:**
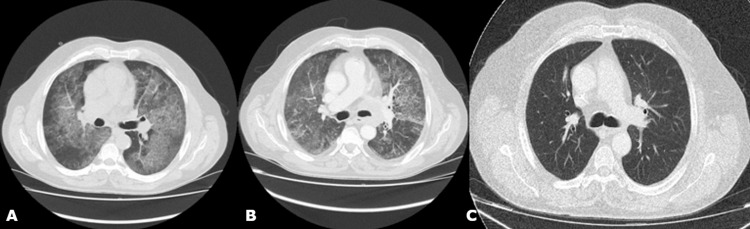
A. Chest computed tomography on admission. B. Radiological improvement three weeks after whole lung lavage and supplementation therapy with inhaled sargramostim. C. Further radiological improvement at six-month follow-up.

A 60-year-old male patient, current smoker (120 pack years), presented to our department with progressive dyspnea on exertion and productive cough with thick white sputum over the past two months. High resolution computed tomography of the chest (HRCT) revealed diffuse ground glass opacities with superimposed interlobular and intralobular septal thickening mainly in the middle and lower lobes, compatible with crazy paving pattern, with slightly enlarged (1.5 cm) left lower paratracheal lymph node (station 4L) that was firstly considered as reactive lymphadenopathy (Figure 1A). He denied the presence of fever, night sweats or weight loss. The patient reported a medical history of arterial hypertension under control receiving irbesartan. Occupational or environmental fibrogenic exposures were not mentioned.

Physical examination revealed the following vital signs: blood pressure of 140/80 mm Hg; heart rate, 80 beats/min; temperature, 36.8˚C and oxygen saturation, 87% on ambient air. Lung auscultation revealed bilateral inspiratory crackles, while heart and abdomen examination was unremarkable. There were no palpable lymph nodes, rash or clubbing.

At the time of referral to the Respiratory Department, arterial blood gases on ambient air (partial pressure of oxygen (pO2): 56 mmHg, partial pressure of carbon dioxide (pCO2): 35 mmHg, pH: 7.41, bicarbonate (HCO3): 23 mmol/L) were indicative of respiratory failure type 1. The complete blood and metabolic panel as well as immunologic and serology tests (anti-nuclear antibodies (ANA), cytoplasmic antineutrophil cytoplasmic antibodies (c-ANCA), perinuclear antineutrophil cytoplasmic antibodies (p-ANCA), rheumatoid factor (RF), anti-cyclic citrullinated peptide (anti-CCP)) were normal, except for positive antibody transcriptional intermediary factor-γ1 (TIF-1γ) in myositis panel that was considered cross-reactivity and raised the suspicion of autoimmune PAP. Subsequently, the patient underwent conventional bronchoscopy without abnormal structural findings. Culture of washing for common pathogens or Mycobacterium infection and cytological examination of washing for malignancy were negative. Analysis of bronchoalveolar lavage (BAL) revealed milky appearance, mild lymphocytosis, mild neutrophilia (macrophages: 52%, lymphocytes: 28%, neutrophils 19%, eosinophils: 1%) and positive periodic acid-Schiff (PAS) stain. Histopathological examination of the transbronchial biopsy of lateral and posterior basilar segmental bronchi (LB9-10) was compatible with inflammation, with no signs of malignancy. Pulmonary function tests (PFTs) showed moderate impairment of diffusing capacity of the lungs (forced vital capacity (FVC): 3.46, 89%, forced expiratory volume in the first second (FEV1): 2.68, 88%, FEV1/FVC: 0.77, diffusing capacity of lung for carbon monoxide (DLCO): 11.85, 50%). Echocardiogram was normal with no signs of pulmonary hypertension.

On the basis of compatible clinical (cough and dyspnea on exertion), functional (diffusing capacity impairment) and radiological features (crazy paving), a working diagnosis of alveolar pulmonary proteinosis was set. In this context, serum levels of GM-CSF autoantibodies were measured. Titer of anti-GM-CSF antibodies was positive in high detectable levels (163 pg/mL) and as a consequence a diagnosis of aPAP was established.

**Figure 2:**
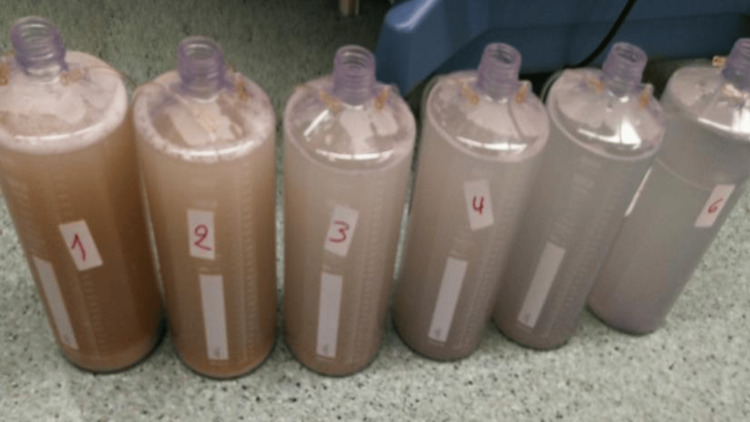
Whole lung lavage.

Following multidisciplinary discussion and taking into account disease severity, whole lung lavage (WLL) -the standard of care- was promptly implemented in a total of four cycles (twice for each lung) (Figure 2). Due to persistent life-threatening respiratory failure, immunosuppressive therapy with rituximab and high doses of corticosteroids in tapering were additionally implemented a few days later and subsequently, the patient underwent three cycles of plasmapheresis (each cycle included five sessions) in order to diminish the concentration of anti-GM-CSF serum antibodies. During hospitalization the patient was supported with supplemental oxygen therapy through a high-flow nasal cannula system. Finally, when the patient clinically improved, we commenced supplementation therapy with inhaled sargramostim (recombinant GM-CSF) 125 μg twice daily.

**Figure 3:**
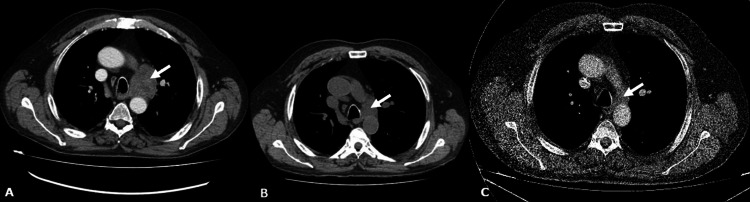
A. Enlargement of lower left paratracheal lymph node 4L (4.3cm) at a three-week follow-up chest computed tomography. B. Decrease in size of 4L lymph node following chemotherapy. C. Further decrease in size of 4L lymph node at six-month follow-up. White arrows highlight 4L lymph node.

In a follow-up appointment after three weeks, the patient presented with clinical improvement without episodes of dyspnea or desaturation on exertion. Despite radiological improvement of ground glass opacities and intralobular thickening on HRCT, a new-onset enlargement of lower left paratracheal lymph node 4L (4.3cm) was observed (Figure 3A). Consequently, the patient underwent an endobronchial ultrasound bronchoscopy (EBUS). Histopathological examination of EBUS-guided transbronchial needle biopsy of 4L lymph node was compatible with SCLC. More specifically, histopathological features revealed small cells with scant cytoplasm with nuclear molding and tightly packed small cells with intratumoral necrosis, while immunohistochemistry revealed positive markers of AE3/CK7, TTF-1, CD56, chromogranin and Ki67 at high levels. Positron emission tomography (PET)-CT and brain magnetic resonance imaging (MRI) were negative for any lung lesion or extrapulmonary malignancy. As a consequence, a limited-stage SCLC report was suggested. A combination of chemotherapeutic agents, carboplatin and etoposide, was promptly administrated, on a three-day protocol, based on treatment guidelines for SCLC. One month later, the patient’s HRCT revealed radiological improvement, with decrease in size of the 4L lymph node and in the extent of parenchymal opacities (Figures 1B, 3B). The patient remained at supplementation treatment with inhaled GM-CSF for aPAP and chemotherapy for SCLC without adverse events. Close radiological and functional (PFTs) follow-up was recommended and at six-month follow-up further radiological improvement was noticed (Figures 1C, 3C).

## Discussion

In this report we present a rare case of co-existence of aPAP and lung cancer. Currently there are only a few described cases of coexistence of pulmonary alveolar proteinosis with primary lung cancer [7-11]. Despite advances in understanding the molecular pathogenesis of aPAP through basic and translational research, studies have not clarified the relationship between PAP and primary lung cancer. Previous reports suggested that increased pneumocytic proliferation activity in PAP may be a causative factor for lung development, that elevated number of functionally incompetent macrophages plays a key role in lung carcinogenesis and that lung cancer development exerts a local inhibitory effect on macrophages through the secretion of a chemical immune inhibitor, respectively [12-14]. Nowadays, even though an epidemiological association between PAP per se and lung cancer has not been established, abundant evidence has shown that interstitial lung diseases represent an independent risk factor for lung cancer development. More specifically, pulmonary fibrosis presents with similar characteristics to cancer with regard to absence of proteostasis, immune dysregulation, senescence and resistance to apoptosis, telomere attrition and impaired cellular bioenergetics [15]. Yet, whether there are specific common mechanisms between PAP and lung cancer is still an unmet need and molecular enrichment of future studies in this field could hopefully contribute to the prediction of the risk of carcinogenesis in patients with PAP.

The main pillars of treatment in patients with PAP are based on pathogenetic causes and on disease severity. The current standard of care in primary PAP and some causes of secondary PAP is WLL [3]. With regards to aPAP, inhaled GM-CSF is safe and effective, while other therapeutic approaches including plasmapheresis and anti-B lymphocyte therapy need further research to be established, as they have appeared to have limited efficacy in some studies [5,6,16]. Furthermore, effectiveness of corticosteroid therapy for aPAP has been doubted as corticosteroids can suppress alveolar macrophage function and might worsen disease severity and increase the risk of infections [17]. Nevertheless, in life-threatening cases, combination of therapies has been applied as rescue therapy [18,19]. To this end, the most reliable biomarker of life-threatening disease is MUC1 rs4072037. In particular, Bonella et al. showed that the MUC1 rs4072037 A/A genotype is associated with more severe pulmonary dysfunction and a higher rate of disease progression in patients with PAP [20].

## Conclusions

Based on the above, despite applied treatment modalities, there is an amenable need for close follow-up in management of PAP. Increasing awareness is a crucial step for early diagnosis and optimal management as these patients may develop -except for fibrotic phenotype and respiratory failure- concomitant lung cancer and benefit from timely interventions.
